# The Effect of Pelvic Floor Muscles Exercise on Quality of Life in Women with Stress Urinary Incontinence and Its Relationship with Vaginal Deliveries: A Randomized Trial

**DOI:** 10.1155/2019/5321864

**Published:** 2019-01-06

**Authors:** Magdalena Ptak, Sylwester Ciećwież, Agnieszka Brodowska, Andrzej Starczewski, Jolanta Nawrocka-Rutkowska, Esther Diaz-Mohedo, Iwona Rotter

**Affiliations:** ^1^Department of Medical Rehabilitation and Clinical Physiotherapy, Pomeranian Medical University in Szczecin, Żołnierska 54 str.,71-210, Szczecin, Poland; ^2^Department of Gynaecology, Endocrinology and Gynaecologic Oncology, Pomeranian Medical University, Unii Lubelskiej 1 str., 71-252, Szczecin, Poland; ^3^Department of Physiotherapy, University of Málga, 29071 Málaga, Spain

## Abstract

**Introduction:**

Urinary incontinence (UI) is a health problem affecting the quality of women's lives (QOL) at various life stages. Stress urinary incontinence (SUI) can be caused by previous vaginal deliveries and is especially likely to occur in the perimenopausal period. The most commonly recommended first-choice treatment methods involve exercises for the pelvic floor muscles (PFM). The aim of this study was to assess the impact of isolated PFM exercises and combined training of the PFM and the m.transversus abdominis (TrA) muscle on the QoL of patients with SUI with regard to the number of vaginal deliveries.

**Material and Methods:**

137 women with SUI were qualified for analysis (mean age 53,1 ± 5,5). To assess the effectiveness of PFM training QOL questionnaire was used (ICIQ-LUTS qol). PFM training for groups A (PFM+TrA) and B (PFM) was intended for 12 weeks. Statistica v. 12.0 PL, StatSoft, USA, was used for statistical calculations.

**Results:**

The analysis demonstrated that conservative treatment based on the A training program (PFM + TrA) yielded statistically significantly better results than the B program (PFM), with the improvement observed in such QoL domains as the performance of household duties, physical activity and travelling, social limitations, emotions, sleep problems and fatigue, the frequency of changing panty liners, fluid intake control, and embarrassment.

**Conclusion:**

Both the combined training of the PFM and the synergistic (TrA) muscle and the isolated PFM exercises improve the QoL of women with SUI. Nonetheless, the combined PFM and TrA muscle physiotherapy is more effective. The exercises for the PFM and the synergistic muscle give better results in women who have given birth fewer than three times than isolated PFM exercises.

## 1. Introduction

Urinary incontinence (UI) is a health problem affecting the quality of women's lives at various life stages. According to the data, the incidence of urinary incontinence ranges from 30% to 60%. The International Continence Society (ICS) singles out stress urinary incontinence (SUI), overactive bladder (OAB), overflow incontinence (OI), and functional incontinence (FI). SUI is especially likely to occur in the perimenopausal period, when tissue resilience decreases as a result of lower estrogen levels. This type of urinary incontinence can be caused by previous vaginal deliveries. During natural childbirth, the birth canal tissues are excessively stretched, and damage to the levator ani muscle and the visceral pelvic fascia may happen [1]. Despite the mechanisms of tissue regeneration in this area, researchers indicate reduction abilities of female bodies after three or more deliveries compared with those of nulliparous and women who have given birth only once [2]. Another complication that can arise during vaginal delivery is damage to neurological structures of this area. It can especially happen in the second phase of labor, when the presenting part of the fetus is getting through the birth canal, pressing the nearby nerves. Suspended on the tendinous arch, the levator ani muscle is tightened and stretched. The pubourethral fascia, rectovaginal septum, and perineal body are strained and may even be ruptured. At the end of the second phase of the labor, the pudendal nerve damage is sometimes observed [3]. The study of 384 women conducted by Pereira with the use of surface electromyography (sEMG) demonstrated that the ability of the pelvic floor muscles (PFM) to be properly tensed varies depending on the life stage. The greatest recruitment of the motor units of the PFM is noted in nulliparas, then primiparas, women after C-section, women after vaginal delivery, and finally climacteric and postmenopausal women [4].

The most commonly recommended first-choice treatment methods, especially for stage 1 SUI, involve exercises for the PFM. Many publications emphasize good effects of this type of conservative treatment, thus indicating that it is appropriate noninvasive management of the mildest—stage 1—SUI. Doing these exercises leads to stabilization of the urethra through an increase in muscle mass. Positive results, patients achieve only after about 6-8 weeks of regular exercises and are manifest themselves in a higher assessment of the QoL [5]. Some researchers indicate the possibility of combining exercises for the PFM and the TrA muscle. According to Stapsford, the TrA muscle is a synergistic muscle, showing natural activity during PFM contractions [6, 7].

The International Consultation Incontinence Questionnaire Lower Urinary Tract Symptoms quality of life (ICIQ-LUTSqol) is a questionnaire measuring the QoL of patients with urinary incontinence [8]. The International Urogynecological Association (IUGA), the ICS, and the International Consultation on Urologic Diseases (ICUD) underline that it is important for questionnaires to use clear, simple language so that patients with different perceptions can complete them on their own [9]. The ICIQ-LUTSqol includes questions concerning the influence of urinary incontinence on particular life domains, namely, physical activity, social contacts, sexual contacts, emotional state, and sleep. Other questions concern activities and feelings that patients experience due to urinary incontinence, among them wearing panty liners, fluid intake control, changing wet underwear, and anxiety associated with unpleasant smell. All items are rated on a four-point scale: never or not at all (1 point), a little or sometimes (2 points), often or moderately (3 points), and very or all the time (4 points). The total score ranges from 19 to 76 points. The ICIQ-LUTSqol has been developed on the basis of King's Health Questionnaire (KHQ) [10, 11].

The aim of this study was to assess the impact of isolated PFM exercises, and combined training of the PFM and the TrA muscle on the QoL of patients with SUI with regard to the number of vaginal deliveries.

## 2. Material

A urodynamic test was performed by means of the Libra device (Medical Measurement System B.V. MMS, Enschede, the Netherlands, 2001). From among 300 patients 150 were qualified for the study; based on the urodynamic test results, an interview was carried out with the Gaudenz questionnaire and gynecological examination unambiguously indicating stage 1 SUI. The qualifying examinations for the study were performed at the Department of Gynecology, Endocrinology and Gynecologic Oncology, Pomeranian Medical University in Szczecin.

The criteria for inclusion in the study were stage 1 SUI without urinary urgency, the 45-60 years age-bracket, at least one vaginal delivery, and the patient's written consent to take part in the study. The criteria for exclusion from the study were higher stage SUI, types of urinary incontinence other than SUI, prolapse according to the Pelvic Organ Prolapse Quantification (POP-Q) system, diabetes, age below 45 and above 60 years, no vaginal deliveries, and no patient's consent for inclusion in the study. The project was approved by the Bioethical Commission of the Pomeranian Medical University in Szczecin (decision no. KB0012/142/13 of 30 September 2013).

## 3. Method

To assess the effectiveness of conservative treatment for stage 1 SUI according to Ingelman-Sundberg scale. In this scale the incontinence severity is graded according to the circumstances or physical activities provoking urinary leakage: grade I: urinary incontinence while coughing or sneezing, grade II: urinary incontinence while running or picking up heavy objects, and grade III: incontinence while walking or climbing stairs [12].

The patients (n = 150) were assigned to two groups by a computer draw—group A (n = 75) and group B (n = 75). As the first step, an interview was carried out using the Polish version of the standardized validated ICIQ-LUTSqol. Next, training programs for group A and group B were designed. For both groups intravaginal estrogen therapy was recommended. After three months, the ICIQ-LUTSqol was used again to assess the patients' QoL. In group A, seven patients did not complete the training: four patients underwent transvaginal tape (TVT) surgery, and three patients did not turn up for the follow-up examination. In group B, six patients did not turn up for the follow-up examination. Eventually, 68 patients from group A and 69 patients from group B were qualified for analysis.

The initial and final QoL assessments were performed by means of the ICIQ-LUTSqol, developed on the basis of the KHQ. This standardized validated questionnaire includes questions concerning such spheres of life as the performance of household duties and outside home activities (Q3), physical limitations (Q4a), social limitations (Q4b), interpersonal limitations (Q5), emotions (Q6), languidness and vitality associated with urinary incontinence (Q7), the performance of such activities as: changing panty liners, restricting fluid intake, changing wet underwear (Q8), and embarrassment (QW). When completing the questionnaire, patients tick one of the four answers: not at all (1 point), a little (2 points), moderately (3 points), and very (4 points). The possible scores range from 19 to 79 points. The scores for this questionnaire were calculated in accordance with the guidelines described in Keller's article, following the example of the base questionnaire—the KHQ—calculation principles [13]. The reliability of the applied survey methods was evaluated by calculating Cronbach's alpha for pre- and posttreatment scores. High pre- and posttreatment values of Cronbach's alpha (0.717 and 0.844, respectively) point to a high reliability of the scale.

The training program for group A (PFM + TrA) was intended for 12 weeks. The exercises were performed four times a week according to the following pattern: three series of 10 repetitions of PFM contractions (6-8 seconds) with the strength of 60-70% MVC (maximum voluntary contraction) and two series of 10 repetitions of PFM contractions with the strength of 30-60% MVC. All contractions were correlated with exhalations and simultaneous TrA muscle contractions and performed in a lying-back position with the legs bent and feet on the ground. Additionally, “the Knack Maneuver” was recommended in case of increased intra-abdominal pressure (IAP) during coughing, sneezing, laughing, and lifting heavy objects. The training program for group B was analogous, but the patients were instructed not to tense the TrA muscle during PFM contractions.

Statistical characteristics of quantitative variables were presented as arithmetic means, standard deviations, medians, minimum, and maximum values as numbers and percentages. Normal distribution of continuous variables was verified with Shapiro-Wilk test. Statistical significance of differences between the study groups was verified with Student t-test and Pearson chi-square test. The effect of number of vaginal deliveries on QOL scores was analyzed with factorial ANOVA: training program (A vs. B) x co-variate (group 0- number of vaginal deliveries <3 vs. group 1- number of vaginal deliveries ≥3), with Tukey post hoc test. All calculations were carried out with Statistica 12 package (StatSoft, USA).* P* < 0.05 was considered statistically significant.

## 4. Results

The original survey developed by the authors was used to collect such characteristics of the study subjects as age, body mass index, number of vaginal deliveries, place of residence, level of physical activity, menopausal status, and smoking (Table 1.).

Table 2 shows the impact of an interfering factor (the number of vaginal deliveries) on the results of conservative treatment applied in women with stage 1 SUI according to the A training program (PFM + TrA) and the B training program (PFM). The significance of differences in the treatment results between the groups was analyzed in order to estimate the impact of this factor.

The analysis results after exercise training demonstrated that conservative treatment based on the A training program (PFM + TrA) yielded statistically significantly better results than the B program (PFM), with the improvement observed in such QoL domains as the performance of household duties and outside home activities (Q3), physical activity and the possibility of travelling (Q4a), and social limitations: interpersonal contacts and the possibility of meeting friends (Q4b), emotions (Q6), sleep problems and fatigue (Q7), the frequency of changing panty liners, fluid intake control, changing wet underwear, anxiety associated with unpleasant smell (Q8), and embarrassment (QW). The analysis of the sum scores of the ICIQ-LUTSqol revealed that a statistically significant change was only reported by the women who had given birth naturally fewer than three times (group 0) and who applied the A training program (PFM + TrA).

## 5. Discussion

The true pelvic floor fulfills numerous functions in a female body. Changes in this area occur not only as a result of a menopause-related drop in hormone levels or hormonal changes in pregnancy, but also a consequence of vaginal deliveries. This may be due to damaged fascias, ligaments, and a part of the peripheral nerves, as well as poor PFM functioning [14]. The comparison between women who have given one natural childbirth and those after one C-section suggests that those delivering vaginally may be at an 8%-12% higher risk of urinary incontinence and reproductive organ prolapse.

We assumed that the number of vaginal deliveries ≥ 3 contributes to the insufficiency of the true pelvic floor, including SUI in the perimenopausal period. This hypothesis is supported by numerous studies describing the connection between urinary incontinence and the number of deliveries. In the study of Lassere et al. [15] the odds ratio for women delivering more than three times was 4.1 and for women delivering only twice 3.0. Also the results obtained by Özdemir et al. [16] are worth mentioning. These authors analyzed 233 women with urinary incontinence in terms of their QoL and the PFM strength. They found that higher numbers of natural childbirths entailed statistically significantly decreased QoL and worse PFM function.

In our study, the numbers of deliveries in the groups were as follows: women after ≥ 3 vaginal deliveries constituted 22% of group A and 17% of group B. The women after < 3 deliveries were substantially more numerous and constituted 78% of group A and 83% of group B.

Tukey's* post hoc* test demonstrated significance only in the group of women who had given birth fewer than three times (group 0). The A training program (PFM + TrA) was significantly more effective than the B training program (PFM) only in this group of women. Similar results were reported by Pereira et al. [4], who analyzed the impact of group PFM exercises (G), individual PFM exercises (I), and exercises performed by the control group (C). The numbers of deliveries in these groups were as follows: 1.46 ± 1.50 (G), 1.26 ± 1.27 (I), and 2.13 ± 1.45 (C). Both group and individual training programs included exercises to be performed in one-hour sessions twice a week for 6 weeks. The exercises were done in a lying-back position, a sitting position, and a standing position. About 100 tonic and phasic PFM contractions were performed during one session. The control group did not do any exercises in this period. Both group and individual exercises resulted in substantial improvement in the performance of household duties and outside home activities (Q3). It should be emphasized that the training was only recommended for the PFM. The analysis of other domains of the KHQ, employed by these researchers, shows that despite a small number of deliveries in the studied groups, significant improvement was only observed in the abovementioned domain and the domains of emotions (Q6), sleep problems (Q7), and the frequency of changing panty liners, restricting fluid intake, and changing wet underwear (Q8).

A significant upturn in the performance of household duties (Q3) was also confirmed by Fitz et al. [17], who assessed effects of the three-month training in 36 women with SUI. The principles of the PFM training performed three times a week were as follows: three sessions of 10 slow contraction repetitions, and 3-4 quick contraction repetitions in a lying-back position, a sitting position, and a standing position. The mean number of deliveries in the study group was 2.5 ± 2.2. The authors demonstrated significant differences in all QoL domains assessed by the KHQ.

The study of Nascimento-Correia et al. [18] involved 30 patients who performed group PFM exercises in a lying-back position and in a sitting position for one hour once a week over a 12-week period. The mean number of deliveries in the study group was 1.47 ± 1.51, and in the control—not exercising—group: 2.13 ± 1.46. In this study, the patients only reported improvement in the performance of household duties (Q3), sleep problems (Q7), and the frequency of changing panty liners, fluid intake control, and changing underwear (Q8).

The results described by Hirakawa et al. [19], who compared the effectiveness of classic PFM exercises and PFM exercises combined with biofeedback therapy in 46 women with SUI, show a different distribution of statistically significant differences in the QoL domains. The patients doing only PFM exercises reported improvement in the ability to perform household duties (Q3), physical activity and travelling (Q4a), and SUI related emotions (Q6), as well as the lesser necessity of controlling fluid intake and changing panty liners (Q8). In this group, the mean number of vaginal deliveries was 2.1 ± 0.6.

Kashanian et al. [20] analyzed a group of 91 women to compare the results of PFM exercises and PFM exercises performed using the “Kegelmaster” device. The authors employed the Incontinence Quality of Life (I-QOL) questionnaire and the Urogenital Distress Inventory (UDI) for general QoL assessment after completed treatment. The study was carried out over 12 weeks, during which the patients performed 6-8-second PFM contractions with a 6-second break for 15 minutes twice a day. The mean numbers of births in the groups were 3.56 ± 1.95 and 3.20 ± 1.00 respectively. Kashanian et al. reported the general QoL improvement after completed conservative treatment in the patients doing isolated PFM exercises. A significant upturn was confirmed by two questionnaires.

In their investigation, Kim et al. [21] performed the Valsalva maneuver in three groups: nulliparous, women who had given birth naturally, and women who had a C-section. The authors performed ultrasound measurement of the muscle thickness, simultaneously assessing intravaginal pressure by means of a perineometer. The examination demonstrated substantial differences in the TrA muscle thickness during pushing between the three groups. The thickness of this muscle was smallest during pushing in women after natural childbirth. There was also a significant difference in the external abdominal oblique muscle between the groups. The authors claim that low intravaginal pressure after natural childbirth and after C-section confirms that pregnancy and labor contribute to the ability of TrA to contract. This conclusion may suggest that the greater number of pregnancies, and thus natural childbirths, worsens the functioning of the TrA muscle and contributes to the effectiveness of the PFM + TrA training program. The results presented in our study show that in the case of patients who have delivered fewer than three times, the A training program (PFM + TrA) is significantly more effective than the B training program (PFM). This confirms that the number of deliveries contributes to the effectiveness of the training.

## 6. Conclusions

Both the combined training of the PFM and the synergistic (TrA) muscle, and the isolated PFM exercises improve the QoL of women with SUI. Nonetheless, the combined PFM and TrA muscle physiotherapy is more effective. The exercises for the PFM and the synergistic muscle give better results in women who have given birth fewer than three times than isolated PFM exercises.

## 7. Limitations

The study involved 137 patients. Considering a huge number of women who experience SUI, it is necessary to conduct further research on their QoL levels. In the future, the strength of the PFM could be assessed using a perineometer—an instrument for measuring intravaginal pressure produced by PFM contractions.

## Figures and Tables

**Table 1:** The features of group A (PFM + TrA) and group B (PFM).

		**Group A** **n=68** **PFM + TrA**	**Group B** **n=69** **PFM**	p
Age ($\overset{-}{x}$+SD, years)		53,2 ± 5,4	53,1 ± 5,6	0,813*∗*

BMI ($\overset{-}{x}$+SD, kg/m^2^)		27,3 ± 4,7	27,3 ± 5,1	1,0*∗*

Number of vaginal deliveries	Group 0 (<3)	53	57	0,492
	Group 1 (≥3)	15	12	

Place of residence (%)	city	74,6	76,2	0,842
	village	26,4	23,8	

Physical activity (%)	sitting	11,9	17,7	0,616
	active	31,1	26,5	
	mixed	57,0	55,8	

Menopausal status (%)	before	47,1	61,4	0,09
	after	52,9	38,6	

Smoking (%)	yes	13,9	12,4	0,8
	no	86,1	87,6	

**Table 2:** The effect of pelvic floor muscles exercise on quality of life in group A (PFM + TrA) and B (PFM). The relationship with number of vaginal deliveries—results of post hoc test after exercise training.

Score	groups	NVD Group 0 (<3) Group 1 (≥3)	$\overset{-}{x}\pm SD$	test *post hoc* Tukey
Q3	A	0	18,4 ± 20,1	A_0_ vs B_0_ p=0,025 A_1_ vs B_1_ p=0,789 A_0_ vs A_1_ p=0,935 B_0_ vs B_1_ p=0,964
		1	11,1 ± 19,2	
	B	0	30,2 ± 20,1	
		1	33,3 ± 21,1	

Q4a	A	0	19,9 ± 15,1	A_0_ vs B_0_ p=0,010 A_1_ vs B_1_ p=0,442 A_0_ vs A_1_ p=0,892 B_0_ vs B_1_ p=0,807
		1	11,1 ± 9,6	
	B	0	32,8 ± 20,9	
		1	41,7 ± 25,3	

Q4b	A	0	6,6 ± 9,8	A_0_ vs B_0_ p<0,001 A_1_ vs B_1_ p=0,848 A_0_ vs A_1_ p=0,999 B_0_ vs B_1_ p=0,313
		1	11,1 ± 0,0	
	B	0	21,5 ± 15,8	
		1	27,8 ± 25,1	

Q5	A	0	14,9 ± 25,3	A_0_ vs B_0_ p=0,482 A_1_ vs B_1_ p=0,638 A_0_ vs A_1_ p=0,999 B_0_ vs B_1_ p=0,810
		1	16,7 ± 16,7	
	B	0	24,2 ± 25,5	
		1	22,4 ± 22,1	

Q6	A	0	11,6 ± 14,4	A_0_ vs B_0_ p<0,001 A_1_ vs B_1_ p=0,708 A_0_ vs A_1_ p=0,999 B_0_ vs B_1_ p=0,139
		1	7,4 ± 6,4	
	B	0	27,8 ± 19,6	
		1	29,6 ± 33,5	

Q7	A	0	19,6 ± 20,1	A_0_ vs B_0_ p=0,006 A_1_ vs B_1_ p=0,963 A_0_ vs A_1_ p=0,624 B_0_ vs B_1_ p=0,997
		1	22,3 ± 19,2	
	B	0	33,9 ± 19,5	
		1	38,9 ± 13,6	

Q8	A	0	24,9 ± 17,9	A_0_ vs B_0_ p<0,001 A_1_ vs B_1_ p=0,125 A_0_ vs A_1_ p=0,997 B_0_ vs B_1_ p=0,687
		1	11,1 ± 12,7	
	B	0	42,4 ± 17,5	
		1	48,6 ± 15,3	

QW	A	0	14,9 ± 21,1	A_0_ vs B_0_ p=0,034 A_1_ vs B_1_ p=0,685 A_0_ vs A_1_ p=0,991 B_0_ vs B_1_ p=0,996
		1	11,1 ± 19,2	
	B	0	30,2 ± 26,4	
		1	44,8 ± 23,1	

Suma scores	A	0	116,0 ± 87,6	A_0_ vs B_0_ p<0,001 A_1_ vs B_1_ p=0,111 A_0_ vs A_1_ p=0,999 B_0_ vs B_1_ p=0,970
		1	90,7 ± 13,1	
	B	0	212,8 ± 88,1	
		1	269,9 ± 113,5	

NVD: number of vaginal deliveries (NVD).

group A [NVD = 0 (<3) n = 53; NVD = 1 (≥3) n = 15].

group B [NVD = 0 (<3) n = 57; NVD =1 (≥3) n = 12).

## Data Availability

The data used to support the findings of this study are available from the corresponding author upon request.
